# A Rare Case of Acute Rheumatic Fever Diagnosed After Recent Travel Abroad

**DOI:** 10.3390/idr18040079

**Published:** 2026-07-27

**Authors:** Debora Agreiter, Stefan Fuchs, Franziska Marti

**Affiliations:** 1 Department of Internal Medicine, Hospital Walenstadt, 8880 Walenstadt, Switzerland; debora.agreiter@ksgr.ch; 2 Department of Hematology, Cantonal Hospital of Graubünden, 7000 Chur, Switzerland; 3 Department of Infectiology, Cantonal Hospital of Graubünden, 7000 Chur, Switzerland; franziska.marti@ksgr.ch

**Keywords:** rheumatic fever, arthralgia, *GAS*-pharyngitis, penicillin, secondary prophylaxis

## Abstract

Background: Acute rheumatic fever (ARF) is a clinical syndrome triggered by *group A streptococcal* (*GAS*) infections and characterized by fever, carditis, and arthritis as its main manifestations. Diagnosis relies on the revised Jones criteria. We report a rare case of ARF diagnosed in Switzerland. Case Presentation: An 18-year-old woman presented to the emergency department five days after returning from a one-week stay in Egypt with intermittent fever and a transient sore throat, followed by migratory joint pain. Laboratory testing revealed leukocytosis, elevated C-reactive protein (CRP) and erythrocyte sedimentation rate (ESR). Chest X-ray, urine analysis, and (travel-associated) pathogen-testing (*Epstein–Barr virus*, *Cytomegalovirus*, *human immunodeficiency virus*, *Malaria*, *Dengue*, *Zika*, *Chikungunya*) were unremarkable. Blood cultures remained negative. Initially, a viral respiratory infection was suspected and symptomatic treatment established. Days later the patient was re-evaluated due to persistent symptoms. Leukocyte count (Lc) and CRP further increased and subcutaneous nodules appeared. The antistreptolysin-O (ASO)-titer was elevated more than three times higher than the normal upper limit and proofed serological evidence of a preceding streptococcal infection (*GAS* throat culture had not been performed). ARF was diagnosed according to the Jones criteria. Treatment with amoxicillin and a high dose of acetylsalicylic acid (ASA) led to a rapid clinical improvement. Secondary prophylaxis with depot penicillin (benzathine penicillin G; 1.2 million units; once monthly) was initiated to prevent rheumatic heart disease. Conclusions: This case highlights a rare occurrence of ARF following travel to Egypt, with probable acquisition of *group A streptococcal* infection during the trip. It emphasizes consideration of ARF even in Western countries with low ARF-prevalence if clinical history is suggestive. Early recognition and initiation of antimicrobial and anti-inflammatory therapy is crucial to prevent cardiac involvement. To the best of our knowledge, this represents one of the first cases diagnosed in Switzerland in the past 20 years.

## 1. Introduction

Acute rheumatic fever (ARF) is a clinical syndrome that occurs as an autoimmune, inflammatory response following tonsillopharyngitis with *group A streptococcus* (*GAS*) [1]. Despite ongoing research, ARF pathophysiology remains incompletely understood [2]. Autoimmune processes are thought to be triggered by molecular mimicry between streptococcal antigens (e.g., M-proteins) and tissue antigens (e.g., myosin) [3]. Clinical manifestations are heterogeneous, primarily involving systemic (fever), cardiac (carditis), neurological (chorea), musculoskeletal (arthritis, arthralgia), and dermatological (erythema marginatum, subcutaneous nodules) manifestations [2]. The Jones criteria, which were originally established by T. Duckett Jones in 1944 [4], are used to establish the diagnosis. Improvements in various medical fields, especially cardiology, led to continuous modifications. A final revision was made in 2015 by Gewitz et al. [5] in the form of a statement paper by the American Heart Association (AHA, Table 1). The diagnosis requires at least two major criteria or one major and two minor criteria. In addition, *GAS*-pharyngitis must be confirmed [5].

The incidence rate of ARF has declined sharply in recent years, especially in developed countries. Western Europe, including Switzerland, has achieved near-elimination of ARF with incidence rates ≤ 10 per 100,000 inhabitants [6,7] and an age-adjusted prevalence of rheumatic heart disease of ≤1 per 1000 inhabitants per year [8]. In comparison, the age-standardized prevalence (per 1000) of rheumatic heart disease (RHD) in Northern Africa, including Egypt, was approximately 4 [8] in 2015. Valuable data regarding the ARF incidence of school-age children in Egypt is not available. The disparity is attributed to social and economic changes in Western countries including improved hygiene and access to medical care (antibiotic drugs) as well as reduced household crowding [5,9,10]. Another factor may be modified M-proteins (a surface protein) of *GAS*-strains, which play a role as key virulence factors and may have become less rheumatogenic [11].

Rheumatic heart disease (RHD) is a serious long-term consequence of ARF and the leading cause of cardiovascular morbidity and premature mortality in children and young adults [12]. It claims an estimated 320,000 lives annually [13], with the highest burden in Oceania, Sub-Saharan Africa, and South Asia [14]. Since subclinical carditis (echocardiographically detectable valvulitis without clinical signs) occurs in up to half of all ARF cases, echocardiography is initiated in all ARF patients [15].

Penicillin therapy should initially be administered to eradicate residual *GAS*, even if the throat culture is negative at this point [10]. In addition, high-dose acetylsalicylic acid (ASA) therapy is recommended for symptomatic relief [6,7]. After ARF, there is a high risk of a recurrent episode in case of a new *GAS*-infection. This may be accompanied by the onset or worsening of existing rheumatic heart disease. Therefore, secondary prophylactic antibiotic therapy is undertaken to prevent rheumatic heart disease [16], and to reduce the risk of progression of latent RHD [17]. The AHA recommendations on the implementation and duration of secondary prophylaxis and the appropriate antibiotics are summarized in Table 2 and Table 3.

Uniform guidelines for primary prophylactic therapy of ARF in the case of *GAS*-pharyngitis are lacking, in part due to the widely varying incidence rates across regions. In endemic regions, penicillin-based primary prevention represents a critical public health priority for reducing the burden of rheumatic heart disease [18].

Increasing migration flows and international travel may alter the epidemiological landscape, potentially leading to higher case numbers and prompting reconsideration of primary prophylactic strategies in the future.

## 2. Detailed Case Description

### 2.1. Medical History

An adult female presented to the emergency department with fever and a sore throat for two days. Five days earlier, she had returned from a one-week stay in Egypt. The fever was intermittent, with peaks of up to 39.0 °C.

Apart from fever, no drenching night sweats or unexplained weight loss was reported. Cardiopulmonary assessment revealed exertion-dependent dyspnea while urinary, gastrointestinal and sexual histories were unremarkable. The patient had not received any travel-specific vaccinations. Childhood immunizations were completed according to the Swiss vaccination schedule. The patient had no relevant pre-existing medical conditions or relevant sociodemographic characteristics.

### 2.2. (Abnormal) Clinical Findings

The patient was an 18-year-old female, fully conscious and adequate. Vital signs were notable for mild tachycardia (108 bpm), tachypnoea (23/min) and subfebrile temperature (37.7 °C). Cardiovascular examination revealed clear, rhythmic heart sounds without murmurs. Neurological assessment was normal. Joints were not swollen, though mild tenderness was noted over both wrists and hips.

### 2.3. Laboratory Diagnostics

Hemoglobin levels were within the normal range. Leukocyte count (Lc) was elevated at 16.8 G/L (reference 4–10 G/L) with neutrophilia (14.1 G/L; reference 1.5–7.7 G/L). All other leukocyte subpopulations were within the normal range. C-reactive protein (CRP) was markedly increased at 13.3 mg/dL (reference < 0.5 mg/dL), and erythrocyte sedimentation rate (ESR) was 50 mm/h (reference 1–20 mm/h), indicating significant inflammation. Electrolytes, renal and liver function tests, and cholestasis parameters were all within normal limits.

### 2.4. Further Diagnostic Steps and Subsequent Course

As part of the evaluation for the source of infection, a chest X-ray was unremarkable. Urinalysis showed no evidence of pyuria or bacteriuria, and a nasopharyngeal swab for *influenza* virus, *respiratory syncytial* virus, and *SARS-CoV-2* was negative. Serological testing for *Epstein–Barr virus*, *Cytomegalovirus*, *Coxiella*, *Francisella*, *Brucella*, and *Rickettsia* was also negative, and blood cultures showed no bacterial growth. *Human immunodeficiency virus* testing was non-reactive. Given the initial suspicion of a viral respiratory infection, symptomatic treatment with metamizole and paracetamol was initiated, and the patient was discharged home.

Four days later (16 days after travel to Egypt), she was re-referred from her family physician with persistent undulating fever, and for the first time localized joint pain in the lower back. She still suffered from a sore throat. Laboratory testing revealed a further rise in Lc (19.5 G/L) and CRP (22.7 mg/dL). Extended testing for travel-associated pathogens, including Malaria (thick smear and rapid diagnostic test), Dengue, Zika, and Chikungunya was negative. Rheumatological causes appeared unlikely, with negative antinuclear antibodies and anti-neutrophil cytoplasmic antibodies. The patient was discharged again with symptomatic therapy.

On another subsequent hospital assessment (one month after the travel to Egypt), the patient suffered from disabling arthralgia, this time predominantly in both hips, wrists and ankles. Inflammatory markers were persistently elevated. New (slightly pruritic) subcutaneous nodules, approximately 1 mm in diameter, were noted on the regions of the trochanter major. After consultation with infectious disease specialists, the antistreptolysin-O (ASO)-titer was ordered. At 669 U/mL it was more than three times higher than the normal upper limit (reference < 200 U/mL). Based on the revised Jones criteria [5] for high-risk populations (major criteria: polyarthralgia; minor criteria: fever, elevated CRP) and evidence of a preceding *group A streptococcal* infection by an elevated ASO-titer, a diagnosis of ARF was finally established. Regarding the described subcutaneous nodules, these were considered only a possible manifestation of ARF because of their atypical clinical characteristics. The streptococcal infection was most likely acquired during the patient’s stay in Egypt (high incidence of ARF), even though *GAS* culture had not been performed at that time.

### 2.5. Treatment and Follow-Up

Following initiation of high-dose ASA at 4 g/day and amoxicillin 750 mg three times daily for ten days, the patient experienced a rapid clinical improvement with complete resolution of symptoms. Two weeks after starting therapy, inflammatory markers had decreased significantly (Lc 12.2 G/L; CRP 3.2 mg/dL). Transthoracic Doppler echocardiography (one month after initial symptom onset and at time of treatment onset) revealed no pathological findings; the electrocardiogram showed no abnormalities including PR, QRS, and QTc intervals within the reference range. Cardiac biomarkers (Troponin T, Creatine Kinase as well as Pro-Brain Natriuretic Peptide) were within normal limits, indicating no evidence of cardiac involvement. In the absence of clinical evidence of carditis, no routine serial follow-up cardiac assessment is currently planned. The ASO-titer initially increased slightly (734 U/mL, which may be due to established variability), consistent with a (sub)acute streptococcal infection. Given the high risk of recurrence with new *GAS*-infections, secondary prophylaxis was initiated using intramuscular benzathine penicillin G (1.2 million units) once monthly for up to five years. Four months after the onset of ARF, the ASO-titer decreased to 449 U/mL, and eight months later to 307 U/mL. To date, the patient remains symptom-free.

## 3. Discussion

This rare case describes a patient with ARF following travel to Egypt, one of the first reported cases diagnosed in Switzerland for years. It illustrates the diagnostic difficulties and the need for high clinical suspicion: the patient’s presentation was relatively non-specific, with fever, arthralgia and a sore throat as its main manifestations. This led to the presumptive diagnosis of a viral respiratory infection with reactive arthralgia. In general, differential diagnosis for this symptom-constellation is broad and includes autoimmune and rheumatologic disorders, infectious etiologies (bacterial and viral) and other conditions which are summarized and described in Table 4.

Our patient presented with disabling polyarthralgia, which is a major Jones criterion for ARF in high-incidence countries like Egypt. Together with fever (≥38 °C) and CRP ≥ 3.0 mg/dL as two minor criteria, the diagnosis of definite ARF could be made. Importantly, evidence of a preceding *group A streptococcal* infection was provided by an elevated ASO-titer of 669 IU/mL.

The manifestation (pruritic) of the subcutaneous nodules is not typical for subcutaneous nodules in ARF, as they are most commonly non-pruritic [5]. Therefore, it is not clear if this manifestation should be labeled as a second major criteria according to the revised Jones criteria.

Next to the need for clinical suspicion, a thorough travel history and its temporal association with symptom onset was crucial for diagnosis. The initial infection with *GAS* is thought to be travel-related, as Egypt has a significantly higher prevalence of ARF/RHD compared to Switzerland [6,7], even though it cannot be ruled out that the initial transmission happened in Switzerland. Therefore, this case also highlights the impact of increasing global mobility and migration on the epidemiology of *GAS*-infection and ARF incidence in low-prevalence Western countries.

While the literature extensively documents ARF and the remaining burden of RHD in immigrants from endemic regions to developed countries [8], it does not specifically address whether these cases represent infections acquired in the country of origin versus the destination country, nor does it describe travel-associated ARF in short-term travelers. Therefore, this case represents an unusual presentation pattern—ARF diagnosed in a developed country (Switzerland) in a patient with a recent travel history to an endemic region (Egypt).

Our patient showed a rapid clinical improvement after initiation of high-dose ASA and penicillin. This response and the progressively declining ASO-titer (following a short initial increase consistent with (sub)acute infection) supported the diagnosis. Testing for *GAS*-pharyngitis with a pharyngeal swab was missed at initial presentation, which limits diagnostic certainty. Nevertheless, due to the clinical presentation, the rapid symptom remission after initiating NSAID-therapy as well as the ASO-titer dynamics, the diagnosis of ARF is highly likely, even though we do not have microbiological confirmation of an initial *GAS*-infection.

We established secondary prophylactic therapy with depot penicillin to prevent rheumatic heart disease, given the high recurrence rate after a subsequent infection with *GAS* [16]. In accordance with current practice guidelines the patient will receive intramuscular benzathine penicillin G (1.2 million units) once monthly [2]. As there was no clinical, laboratory, electro- or echocardiographic evidence of cardiac involvement, the duration of secondary prophylactic therapy will be up to five years (see Table 2). Even after completion of the five years, antibiotic prophylaxis might be still necessary depending on the individualized risk profile [20].

To date, the patient remains without evidence of a recurrent GAS-infection and is completely symptom-free.

## 4. Conclusions

This case report illustrates that ARF remains an important differential diagnosis in patients with unspecific symptoms such as migratory arthritis/arthralgia, systemic inflammation and a sore throat, even in low-incidence populations, particularly when a detailed travel history is suggestive. Travel-related *GAS* acquisition is highly probable in this case but cannot conclusively be proven.

The diagnosis can be challenging and requires a high level of clinical suspicion and awareness. Antibiotic therapy is essential in confirmed ARF, especially to prevent cardiac complications. Secondary prophylaxis aims to prevent recurrent *GAS*-infections, thereby reducing the risk of recurrent or progressive rheumatic heart disease.

## Figures and Tables

**Table 1:** Revised Jones criteria for acute rheumatic fever (ARF) according to Gewitz et al. [5].

Criteria	Low-Risk Population *	High-Risk Population **
Major Criteria	Carditis (clinical and subclinical)	Carditis (clinical and subclinical)
	Polyarthritis	Mono-/polyarthritis/polyarthralgia
	Chorea	Chorea
	Erythema marginatum	Erythema marginatum
	Subcutaneous nodules	Subcutaneous nodules
Minor Criteria	Polyarthralgia	Monoarthralgia
	Fever (≥38.5 °C)	Fever (≥38 °C)
	ESR ≥ 60 mm/h or CRP ≥ 3.0 mg/dL	ESR ≥ 30 mm/h or CRP ≥ 3.0 mg/dL
	Prolonged PQ interval	Prolonged PQ interval

* Low-risk populations are defined as those with an ARF incidence of ≤2 per 100,000 school-age children or an age-adjusted prevalence of rheumatic heart disease of ≤1 per 1000 inhabitants per year. ** High-risk populations are defined as those with an ARF incidence of >2 per 100,000 school-age children or an age-adjusted prevalence of rheumatic heart disease of >1 per 1000 inhabitants per year. Abbreviations: CRP, C-reactive protein; ESR, erythrocyte sedimentation rate.

**Table 2:** Duration of secondary prophylaxis for ARF [16].

Clinical Setting	Duration Since Last Episode	Level of Evidence
Rheumatic fever with carditis and residual heart disease (persistent valve disease *)	10 years or until the age of 40 (whichever is longer)	IC
Rheumatic fever with carditis without residual heart disease	10 years or until the age of 21 (whichever is longer)	IC
Rheumatic fever without carditis	5 years or until the age of 21 (whichever is longer)	IC

* Clinical or echocardiographic evidence.

**Table 3:** Antibiotic secondary prophylaxis of ARF [16].

Preparation	Dosing	Application	Level of Evidence
Benzathine penicillin G	0.6 million units ≤ 27 kg; 1.2 million units > 27 kg every four weeks *	intramuscularly	IA
Penicillin V	250 mg twice daily	orally	IB
Sulfadiazines	500 mg daily ≤ 27 kg; 1000 mg daily > 27 kg	orally	IB
In case of penicillin and sulfadiazine allergy			
Macrolides or azalides	variable	orally	IC

* In high-risk situations, administration every three weeks is recommended.

**Table 4:** Differential diagnoses for joint pain and recurrent fever (non-exhaustive), including their typical clinical symptoms, underlying pathophysiology, and therapeutic approaches [19].

Diagnosis	Symptoms	Pathophysiology	Therapy
Septic arthritis	usually monoarthritic, arthralgia, fever	bacterial joint infection with systemic seeding	antibiotic therapy, debridement (if necessary), arthrotomy
Gout	acute: mono- to polyarthritis, fever; chronic: soft tissue and bone tophi, urate nephropathy	precipitation of urate crystals, activation of the inflammasome complex	acute: NSAIDs, colchicine, corticosteroids, cryotherapy
Acute rheumatic fever	fever, arthralgia, arthritis, carditis, Sydenham’s chorea, subcutaneous nodules, erythema marginatum	autoimmune reaction in cross-reactions between streptococcal antigens and tissue antigens	penicillin, ASA and corticosteroids, antibiotic recurrence prophylaxis
Rheumatoid arthritis	peripheral polyarthritis, non-specific general symptoms, extra-articular organ manifestations	autoimmune disease induces inflammatory synovialitis	physical therapy, immunosuppressants, biologics, NSAIDs
Lyme arthritis	(mono-) arthritis, other possible manifestations: encephalomyelitis, erythema migrans, meningoradiculitis Bannwarth, myocarditis	*Borrelia burgdorferi sensu lato* infection	Doxycyclin, amoxicillin or Cefotaxim
Systemic lupus erythematosus	polyarthritis, myositis, skin changes, serositis, neurological involvement, lupus nephritis	immunological systemic disease with impaired activity of T and B cells, complement activation	NSAIDs, hydroxychloroquine, corticosteroids, belimumab, MTX, azathioprine, rituximab
Reactive arthritis	Reiter’s Trias: arthritis, urethritis, conjunctivitis, dactylitis, uveitis, general symptoms	post-infectious autoimmune reaction after enteric infection or urethritis	infection remediation, symptomatic: physical therapy, NSAIDs, corticosteroids
Adult Still’s disease	salmon-colored, small- spotted maculopapular rash, fever, myalgias/arthralgias, symmetrical polyarthritis, sore throat, lung and heart involvement	autoimmune disease, exact pathophysiology unknown	steroids, MTX, IL-1 and IL-6 blockade, TNF blockade, immunoglobulins
Arthritis/arthralgia in viral infections	arthritides/arthralgias, general symptoms, virus-specific complaints	infection with *Hepatitis B/C*, *HIV*, *parvovirus B19*, *Chikungunya*, *Zika virus*, *Rubella*	virus-dependent, symptomatic and sometimes specific antiviral therapy

Abbreviations: ASA, acetylsalicylic acid; HIV, human immunodeficiency virus; IL, interleukin; MTX, methotrexate; NSAIDs, nonsteroidal anti-inflammatory drugs; TNF, tumor necrosis factor.

## Data Availability

The original contributions presented in this study are included in the article. Further inquiries can be directed to the corresponding author.

## Abbreviations

The following abbreviations are used in this manuscript:

AHA	American Heart Association
ARF	Acute rheumatic fever
ASA	Acetylsalicylic acid
ESR	Erythrocyte sedimentation rate
CRP	C-reactive protein
GAS	*Group A streptococci*
Lc	Leukocyte count
RHD	Rheumatic heart disease
